# The factor structure the PHQ-9 in Russian patients recovered from COVID-19

**DOI:** 10.1192/j.eurpsy.2024.1051

**Published:** 2024-08-27

**Authors:** M. Zinchuk, G. Kustov, S. Popova, A. Razmakhnin, D. Zhuravlev, R. Akzhigitov, A. Guekht

**Affiliations:** ^1^ Moscow Research and Clinical Centre for Neuropsychiatry; ^2^Pirogov Russian National Research Medical University, Moscow, Russian Federation

## Abstract

**Introduction:**

The nine-item Patient Health Questionnaire-9 (PHQ-9) is the first choice for screening for depression in primary care and other medical settings. The PHQ-9 has been shown to be a reliable and valid measure of depression symptoms, but there is disagreement among researchers about the factor structure of this questionnaire. Recent systematic reviews have found four different factor models of the PHQ-9, with one- and two-factor models being the most common. This discrepancy may be due to linguistic, cultural and clinical differences between the populations studied. The factor structure of the Russian version of the PHQ-9 during the COVID-19 pandemic has not been examined in any study to date.

**Objectives:**

The aim of our study was to determine the factorial structure and internal consistency of the Russian version of the PHQ-9 in COVID-19 survivors.

**Methods:**

Fourteen thousand 725 (female - 11479 (78.0%), age - 18-79 years (M - 47.09, SD - 12.70) participants completed an online survey including the PHQ-9 and an ad hoc questionnaire focusing on sociodemographic and COVID-related characteristics. McDonald’s omega coefficient was estimated to determine the internal consistency of the questionnaire. Exploratory structural equation modelling (ESEM) with weighted least squares mean and variance adjusted estimator and geomin rotation was performed in Mplus 7.

**Results:**

ESEM provided evidence for a three-factor structure of the PHQ-9, representing affective (items 2, 6, 9), anergic (items 1, 3) and somatic (items 3, 5, 7, 8) dimensions of depression. These factors fit the data well (CFI - 0.998; TLI - 0.994; RMSEA (95% CI) - 0.028 (0.024 - 0.032)), better than a single factor (CFI - 0. 955; TLI - 0. 940; RMSEA (95% CI) - 0.089 (0.087 - 0.092)) and two-factor (CFI - 0.985; TLI - 0.971; RMSEA (95% CI) - 0.062 (0.059 - 0.065)). The McDonald’s omega was 0.82.

**Conclusions:**

Our study revealed a three-factor structure of the Russian version of the PHQ-9 in COVID-19 survivors. COVID-19. A high internal consistency of the Russian version of the instrument was confirmed.

**Disclosure of Interest:**

None Declared

